# Agility and change-of-direction speed are two different abilities also during the execution of repeated trials and in fatigued conditions

**DOI:** 10.1371/journal.pone.0269810

**Published:** 2022-06-10

**Authors:** Gianmarco Ciocca, Antonio Tessitore, Harald Tschan

**Affiliations:** 1 Centre for Sports Science and University Sports, University of Vienna, Vienna, Austria; 2 Department of Movement, Human and Health Sciences, University of Rome “Foro Italico”, Rome, Italy; Federation University Australia, AUSTRALIA

## Abstract

Agility and change of direction speed are two different abilities, but no study has investigated if this difference exists also in fatigued conditions, and whether fatigue develops in a different way in a repeated-trial scenario. Fourteen soccer players (age: 17.0 ± 0.4 yrs; height: 176.9 ± 6.5 cm; body mass: 69.2 ± 6.4 kg) competing in a national-level youth league completed in a randomized counter-balanced crossover design a repeated agility protocol (RA) and a repeated change-of-direction one (RCOD), both consisting in performing 20 consecutive repetitions (work:rest ratio 1:5). The 20 repetitions were divided into 4 blocks (each block containing 5 repetitions) for the analysis. Results show that agility and COD are two different abilities both in rest and fatigue conditions: block 1 RA vs 1 RCOD (p < 0.001; ES = 2.02 huge; r = 0.17 poor; r^2^ = 0.03), 2 RA vs 2 RCOD (p < 0.001; ES = 2.3 huge; r = 0.51 fair; r^2^ = 0.26), 3 RA vs 3 RCOD (p < 0.001; ES = 2.38 huge; r = 0.54 fair; r^2^ = 0.29), and 4 RA vs 4 RCOD (p < 0.001; ES = 2.7 huge; r = 0.41 fair; r^2^ = 0.17). However, the fatigue development in both conditions was similar, with a percentage decrement score (S_dec_) of 7.5% for RA, and 7.3% for RCOD. Ratings of perceived exertions (RPE) were similar too (7.3 ± 1.7, and 6.6 ± 1.9, for RA and RCOD, respectively). However, a significant fatigue-related performance impairment arose earlier in RA (block 2) than in RCOD (block 3). Total RA and total RCOD times were significantly different (p < 0.001; ES = 2.65 huge; r = 0.41 fair; r^2^ = 0.17), suggesting that they are two different and independent abilities.

## Introduction

Sport is far more than merely the result and expression of physical skills and capabilities. Sport performances consist of the constant and mutual interaction of athletes’ emotional, psychological, cognitive, physiological, biomechanical, and tactical abilities [1–4].

Accordingly, authors have suggested that situational sport represents an extremely demanding cognitive activity, and that it can be seen as the *“brain’s biggest challenge”* [5, 6]. For instance, in situational team sports like soccer, players are required to manage and collect the most relevant information from a chaotic, space-time pressured and continuously changing environment, to make the quickest and most accurate decision about what (declarative knowledge) and how (procedural knowledge) to do, and finally to perform the action while still monitoring the environment, in order to be able to adjust said action [7–9].

Handling such difficult situations and making complex decisions while being intensively physically active for a long time, may also make soccer a mentally fatiguing activity [6, 10–12], acknowledging the multifaceted nature of fatigue, beyond the only physiological aspects. This would be in line with the definition of mental fatigue, that is a psychobiological state caused by prolonged periods of demanding cognitive activity, which may result in an acute increase in subjective ratings of fatigue and/or an acute decline in several different aspects of performance, (cognitive, physical, or both) [13–16].

The above considerations emphasize the importance of defining, studying, and analyzing sport-specific skills that reflect not only physical factors, but include also perceptual and cognitive components during their execution. For this reason, in 2006, Sheppard and Young [17] proposed as new definition of agility “*a rapid whole-body movement with change of velocity or direction in response to a stimulus*”, highlighting the presence of cognitive and decision-making factors in agility, contrary to COD (Change of Direction) that is just the physical component of it. Thus, even though some authors still use the term *planned agility* to refer to COD ability (without external stimuli) and *reactive agility* for tasks that include a response to stimuli, in our paper we will use respectively the terms COD and agility, according to their original definition which makes redundant or erroneous the use of *planned* and *reactive* terms when referring to agility [18].

Since then, several studies have analyzed the differences between agility and COD, confirming that they are two different and independent skills due to the presence of decision-making factors in the former, thus requiring two specific trainings [18–22]. Research has also shown that agility tests better discriminate between higher and lower-level players than COD tests, highlighting the importance of perception, decision-making, anticipation, and tactical knowledge in situational sports [18, 22, 23]. Moreover, among the agility tests, Paul et al. [23] summarize that more ecological and sport-specific stimuli like human or video better discriminate higher and lower-level players than easier general stimuli like lights. The reason behind this is that perceptual and decision-making skills required during a game are very complex and dynamic in nature, and come from multiple interacting sources. Therefore, using a test that does not resemble such complexity, but just relies on a simpler choice (i.e.: left/right, go/no-go, according to a light stimulus), does not allow a skilled player to use its better ability of retrieving and selecting the most important kinematic cues from the game contest, and to benefit from its own superior knowledge, comprehension, and anticipation skill of the situations, to then select the most appropriate response [23].

Scanlan et al. [24] supported the importance of cognitive and decision-making factors in agility showing their higher relationships with it, in respect to morphological, sprint, and COD speed measures. Likewise, Naylor and Greig [25] showed that cognitive accuracy during the psychological Stroop word-color test was the factor with the strongest relation to agility, followed by anthropometric and eccentric hamstring strength.

However, in situational team sports like soccer, more than the single best performance, the ability to perform repeated efforts with only partial recoveries, then under the influence of fatigue, is the real crucial aspect, since players are required to resist it and to keep on playing in such condition. For this reason, research has focused on tasks with high intensity efforts interspersed with short recovery periods, like the repeated sprint ability (RSA) [26–28], the repeated change of direction (RCOD) ability [29–32], and very few also on repeated agility (RA) ability [33, 34], even if a definition of this term is still missing. Moreover, the work:rest ratios of these repeated tasks, aimed to test players’ ability to perform several high intensity activities resisting to fatigue, typically range from 1:2 to 1:6, also according to the sport practiced, but a 1:5 ratio is usually adopted [26, 28, 31, 35].

According to Altmann et al. [35], comparing and interpreting fatigue measures between such studies is not easy, and caution should be paid because of the large number of different indexes used to analyze the fatigue-related decrement scores, and the different spatial configurations of the physical tasks (distances, turns’ degrees of angles, etc.) [26]. For instance, Ruscello et al. [31] reported a performance decrement of 5.76% in RCOD ability using the index of fatigue (IF%) as a measure (see [26] for direct comparison between methods and formulas) and adopting a 1:5 work:rest ratio. On the contrary, Matlák et al. [33], using always a 1:5 work:rest ratio, but with a very different spatial configuration (less distance per repetition, and different turn angles), reported no clear trend of fatigue during one RA protocol. Furthermore, Madueno et al. [32] showed that active recoveries between RCOD repetitions causes superior physiological stress, leading to a higher performance decrement, compared to passive recoveries.

Nonetheless, while only one study directly analyzed the differences and relationships between RSA and RCOD ability [36], to the best of our knowledge, no one directly compared RCOD to RA to investigate whether agility and COD abilities differ also in a multiple repetition setting, and if fatigue differently develops in these 2 types of tasks. As previously mentioned, in the same way agility and COD ability differ in rest conditions because of the presence of decision-making factor in the former, we hypothesized and expected that this difference persisted also in RA and RCOD (i.e.: under fatigue conditions) for the same reason. Moreover, even with a high discrepancy among studies’ results, research has also shown that physical fatigue raised by short high-intensity exercise bouts may impair perceptual and simple rapid decision-making abilities (e.g.: reacting to light stimuli), while exercise at moderate intensity does not [37]. Labelle et al. [38] showed that physical fatigue raised by high-intensity exercise impaired more complex perceptual-cognitive tasks, especially in lower fit individuals. However, this is in discordance to Royal et al. [39], who demonstrated that high physical exertion improved declarative decision-making in a tactical video-test.

Therefore, the aim of this study was to investigate if performance follows a different trend during a RCOD protocol and a RA one, if fatigue appears and develops differently, and consequently, if agility and COD also differ in fatigued conditions as hypothesized.

## Materials and methods

### Experimental approach to the problem

To investigate these research questions, participants performed RCOD and RA conditions during sessions on two different days in a randomized counterbalanced crossover design. To have the same movement pattern in both conditions (with the only difference being the external stimulus to respond to for the agility condition), a test that has been reliably applied to assess both COD and agility was chosen [40–42].

### Subjects

Fourteen (14) soccer players (age: 17.0 yrs ± 0.4; height: 176.9 ± 6.5 cm; body mass: 69.2 ± 6.4 kg) competing in a national-level youth league took part in the study. Participants had no previous muscular injures within the 60 days before the testing sessions. All subjects and the legal tutors of minor participants were informed about the possible risks and signed an informed consent form before proceeding to any physical test. The study was designed in fulfillment of the ethical guidelines communicated in the Declaration of Helsinki and approved by the University of Vienna Ethics Review Board (Reference Number: 00451).

### Procedures and measurements

Participants underwent two familiarization sessions to be habituated to all tests and procedures. After that, they performed both experimental protocols (RCOD and RA) in a randomized counterbalanced crossover design. Measurements were taken during two experimental sessions (day 1 and 2) interspersed by 6 days, at the same time each day. Participants were instructed to not participate in vigorous exercises 48 hours before the tests. All the tests were delivered on an artificial-grass soccer field where participants used to have trainings and matches. Humidity, wind, temperature, and light conditions did not differ noticeably in the two testing days. Participants were wearing their usual training shoes and equipment.

**Fig 1:**
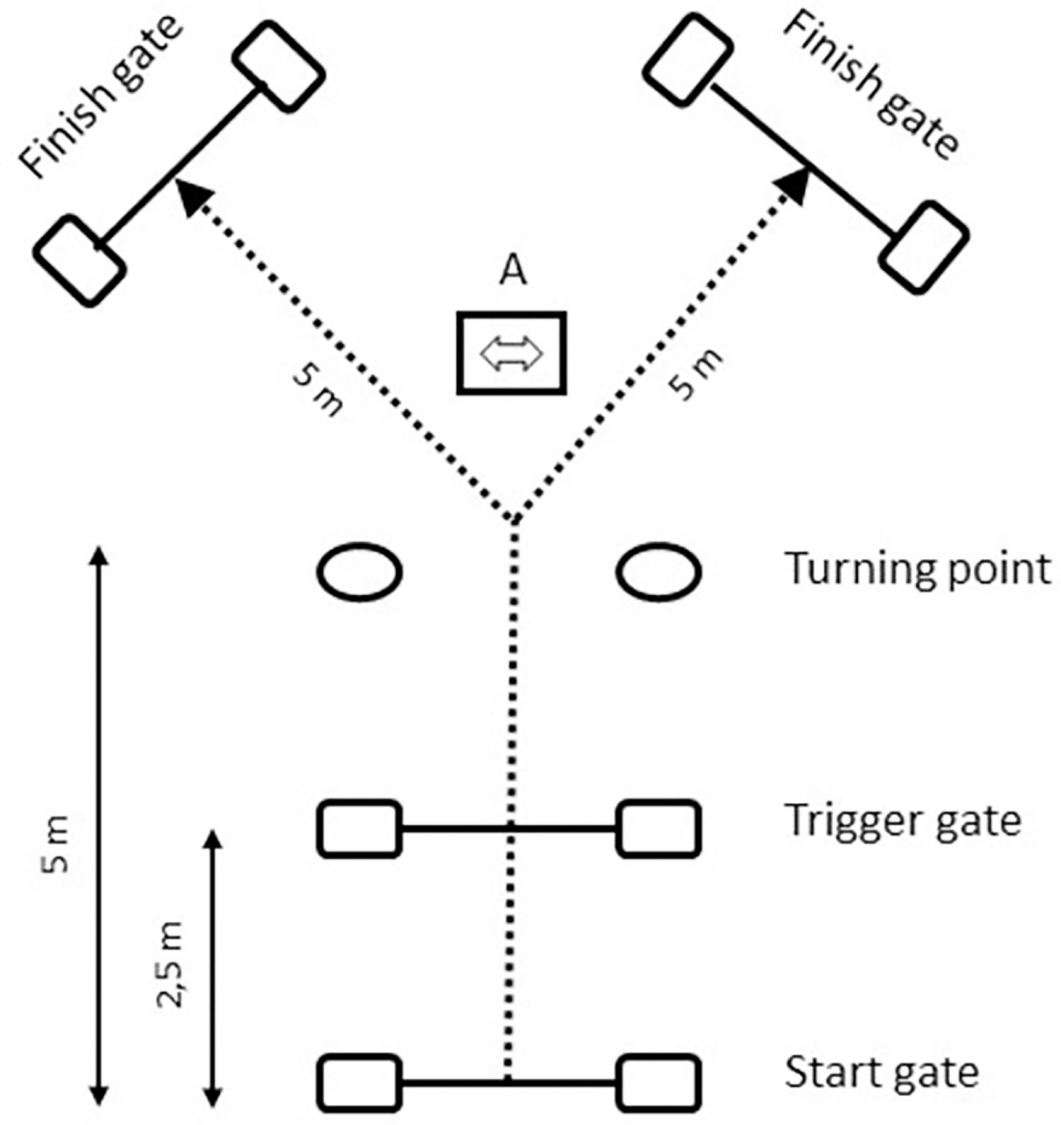
Y-shaped test used for the RCOD and RA protocols. For RA, a LED indicator showing the direction (A) was placed 2 m beyond the turning point, while it was not present in RCOD.

Before each test, subjects performed a 10-minute warm-up consisting in jogging, dynamic functional movements, jumps, sprints, decelerations, and changes of directions, gradually increasing the intensity up to the maximum [36]. After 2 minutes of recovery, the RCOD or RA tests began. The test chosen was a Y-shaped one, which was already shown to be reliable for both COD and agility assessments [40–42]. Participants had to start 30 cm behind the starting line, sprint for 5 m straight, then perform a 45° change of direction to the left or to the right, and to finally sprint for a further 5 m until the finish line (Fig 1). All electronic timing gates were placed at a height of 0.5 m, and 1.5 m width. In the RA test, participants had to change direction to the left or to the right according to a light stimulus that appeared on a LED indicator in front of them (randomly showing an arrow pointing left/right) once they crossed a trigger gate placed 2.5 m after the starting line [42]. Instead, in the RCOD test, subjects were randomly told by voice before the start where they had to turn (left/right). For both conditions, participants had to perform the test 20 consecutive times, with 10 s of recovery between repetitions. This recovery time was chosen to adopt a 1:5 work:rest ratio, since each single repetition took approximately 2 s. After the completion of each repetition and during the 10-second recovery time, participants had to walk/jog to the starting line to prepare and start in time the following trial. For the repetitions of both agility and COD, the number of times players cut per side was randomized (for each repetition, participants had to randomly cut left or right, with a 50% of chance), but not counterbalanced, meaning that a side could be performed more than the other one. That is because the system used in this study (Microgate WittySEM, Microgate, Bolzano, Italy) did not allow to choose and pre-set the sequence of stimuli. However, because of the high number of repetitions performed (20 × 14 = 280, for each condition) and according to manufacturer’s simulations, it was presumed and expected that the total number of cuts per side counterbalances itself close enough to the 50%.

Although Paul et al. [23] have shown that agility tests with light stimuli are less precise in discriminating between higher and lower skilled players (validity) and the less ecological, we chose such a test because we needed it to be reliable for both agility and COD, with as few differences as possible between the executions of the two tasks. A human stimulus (field test) would have increased such differences, and a video stimulus represents a laboratory setting that would have reduced the on-field practical applications. Moreover, as reported by Paul et al. [23], Y-shaped light tests are still the most adopted as testing tools, because of their easy field-applicability and higher reproducibility. Also, since this is the first study directly assessing and comparing the evolution of agility and COD during repeated trials and also in fatigued conditions, it appeared reasonable choosing the most used available test [21, 23]. Finally, discriminating lower and higher skilled players was not an aim of this study.

Times of RCOD and RA trials (from the starting gate to the finish one) were recorded using the Microgate WittySEM system (Microgate, Bolzano, Italy).

At the end of the 20 repetitions, the RPE questionnaire (Borg CR10) was given to participants to assess their ratings of perceived exertion.

### Statistical analysis

Statistical analyses were performed using SPSS software (Version 26.0, SPSS Inc., Chicago, IL, USA). Data are presented as mean ± SD. Cohen’s d was used to calculate effect sizes (ES), and results were interpreted as follow: 0.01–0.2 very small, 0.21–0.5 small, 0.51–0.8 medium, 0.81–1.2 large, 1.21–2.0 very large, > 2.0 huge [43]. The level of significance was set at p ≤ 0.05, with all calculations based on a 95% confidence interval (CI). Shapiro-Wilk test was applied and showed that data were normally distributed. Moreover, a posteriori (“post hoc”) power analysis calculated after the study using the G*Power software (Version 3.1.9.7) and the relative guidelines by Faul et al. [44], confirming the strength of the statistical power (1 – β) and the appropriateness of the sample size.

For the analysis, due to the high number of random (left/right) repetitions (n = 20 for each of the 2 conditions) and the related high variability that may exist between one repetition and the previous/following one, we divided them into 4 blocks (for both RCOD and RA): 1^st^ block for repetitions 1–5, 2^nd^ block for repetitions 6–10, 3^rd^ block for repetitions 11–15, and 4^th^ block for repetitions 16–20. Therefore, each block represents the mean of 5 repetitions. This block organization was also adopted by Matlák et al. [33] in their study on repeated agility. By doing so, it would be possible to see a clearer performance trend between the initial (no fatigue) and final blocks (fatigue), avoiding the risk of an inter-repetition high variability. A second advantage of using this approach is overcoming the inconsistencies that exist among different studies and their respective methods to measure fatigue scores [26, 35], by calculating the performance decrement just as a % difference between the 1^st^ and the following blocks.

A two-way (2 × 4) repeated measures ANOVA was applied (condition [RCOD, RA] × time [1^st^ block, 2^nd^ block, 3^rd^ block, 4^th^ block]) to investigate if performance differently develops in the 2 conditions. The Mauchly’s test was carried out and Greenhouse-Geisser correction was applied if sphericity was violated. One one-way repeated measures ANOVA was then separately applied for the RA condition alone, and one for the RCOD alone, with relative post-hoc when necessary. As mentioned, decrement of performance over the execution of repetitions was calculated as % difference between the 1^st^ block and the following ones, but also using the percentage decrement score (S_dec_) shown in [26], which considers the decrement among all repetitions.

To investigate if agility and COD abilities differ in recovery (1^st^ block) but also in fatigued status (following blocks), 4 separate paired samples t-tests were applied to assess differences between corresponding blocks of the 2 conditions (1^st^ block RA vs 1^st^ block RCOD, 2^nd^ block RA vs 2^nd^ block RCOD, 3^rd^ block RA vs 3^rd^ block RCOD, 4^th^ block RA vs 4^th^ block RCOD). Paired samples t-tests were also applied to assess differences between RCOD and RA total times (the sum of all 20 RCOD and RA repetitions, respectively), and for the RPE values between the RCOD and RA conditions. All correlations were calculated using Pearson’s r and its relative coefficient of determination (r^2^), and the strength of relationships were interpreted as previously suggested [45].

The Kendall rank correlation coefficient (Kendall’s tau) was used to analyze correlations between players’ ranks (1^st^ to 14^th^) in RA and RCOD.

## Results

Blocks’ mean times, and % differences between each block and the same-condition’s first one are reported in Tables 1 and 2 for RA and RCOD, respectively.

**Table 1:** Mean times (s) ± SD of RA blocks, and % difference between each block and the 1^st^ one.

Block	Mean time (s)	Δ (%)
1	2.253 **±** 0.08	-
2	2.321 **±** 0.08 *	3.0
3	2.369 **±** 0.07 *	5.1
4	2.382 **±** 0.07 * §	5.7

* = different from block 1 (p < 0.05); § = different from block 2 (p < 0.05)

**Table 2:** Mean times (s) ± SD of RCOD blocks, and % difference between each block and the 1^st^ one.

Block	Mean time (s)	Δ (%)
1	2.081 **±** 0.09	-
2	2.113 **±** 0.1	1.5
3	2.164 **±** 0.1 *	4.0
4	2.149 **±** 0.1 *	3.3

* = different from block 1 (p < 0.05)

**Fig 2:**
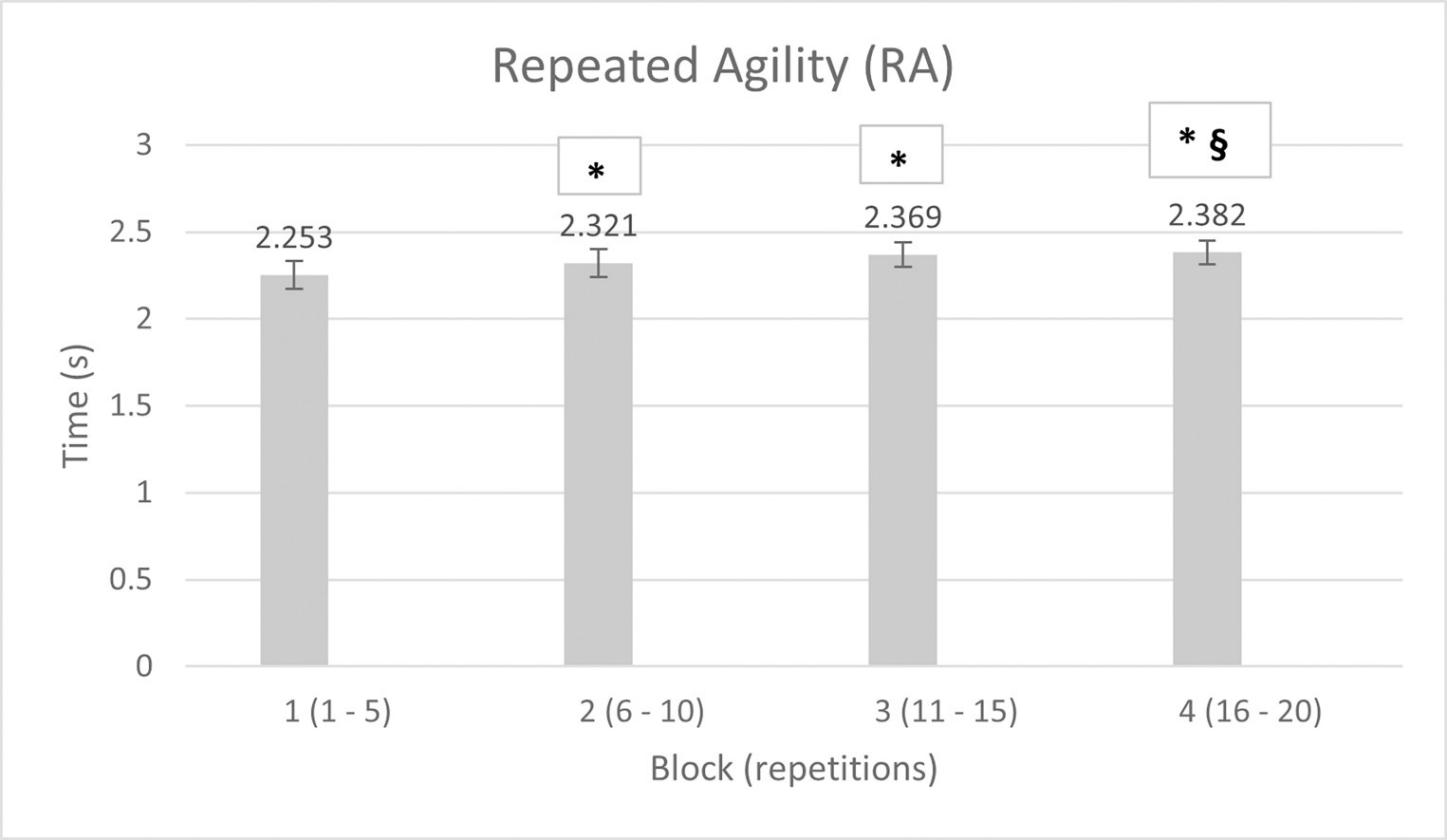
Mean times ± SD of RA blocks. * = different from block 1; § = different from block 2 (p < 0.05).

**Fig 3:**
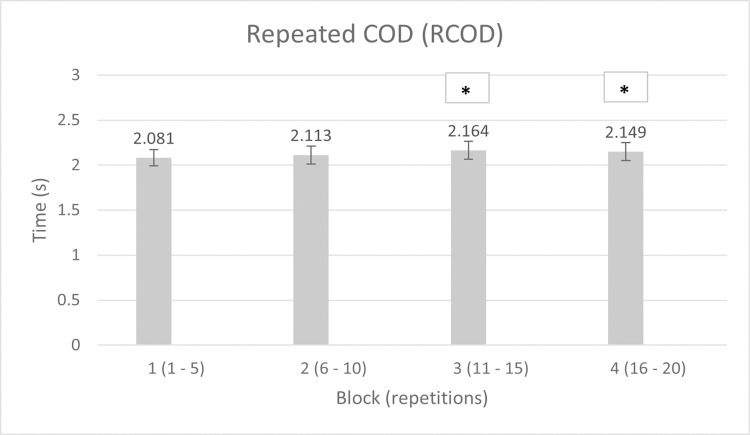
Mean times ± SD of RCOD blocks. * = different from block 1 (p < 0.05).

The two-way (2 × 4) repeated measures ANOVA reported no statistical significance for the interaction condition × time (p = 0.053; η_p_^2^ = 0.177). Instead, the one-way repeated measures ANOVA for the RA blocks revealed significant differences (p < 0.001; η_p_^2^ = 0.651). Respective post hoc showed differences in blocks 1 vs 2 (p = 0.001; ES = 0.85 large), 1 vs 3 (p < 0.001; ES = 1.5 very large), 1 vs 4 (p < 0.001; ES = 1.7 very large), and 2 vs 4 (p = 0.027; ES = 0.81 large) (Fig 2), revealing the fatigue development over the repeated trials. The percentage decrement score (S_dec_) among agility repetitions was 7.5%. The one one-way repeated measures ANOVA for the RCOD blocks revealed significant differences too (p = 0.002; η_p_^2^ = 0.392). Respective post hoc showed differences in blocks 1 vs 3 (p = 0.008; ES = 0.87 large), and 1 vs 4 (p = 0.041; ES = 0.71 medium) (Fig 3), unveiling once again the fatigue development over the repeated trials. The percentage decrement score (S_dec_) among COD repetitions was 7.3%.

**Fig 4:**
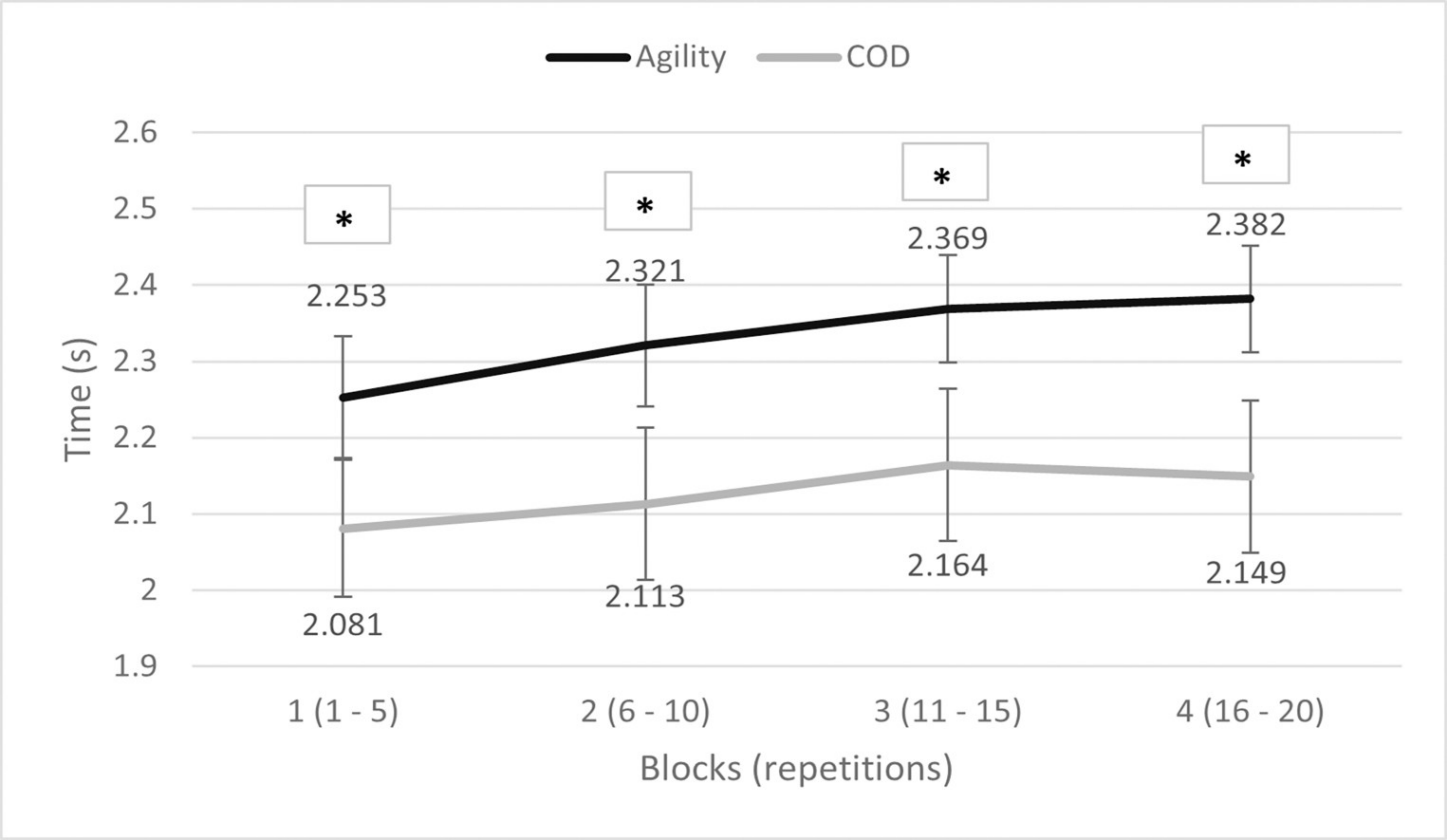
Comparison between RA and RCOD (mean times ± SD). * = different from the corresponding block of the other condition (p < 0.05).

A comparison between the performance decrement among blocks for RA and RCOD is shown in Fig 4. Individual data for RA and RCOD are shown in S1 Fig.

The t-tests and correlation analysis between corresponding blocks of the 2 conditions (RA and RCOD), revealed all significant differences and values: block 1 RA vs 1 RCOD (p < 0.001; ES = 2.02 huge; r = 0.17 poor; r^2^ = 0.03), 2 RA vs 2 RCOD (p < 0.001; ES = 2.3 huge; r = 0.51 fair; r^2^ = 0.26), 3 RA vs 3 RCOD (p < 0.001; ES = 2.38 huge; r = 0.54 fair; r^2^ = 0.29), and 4 RA vs 4 RCOD (p < 0.001; ES = 2.7 huge; r = 0.41 fair; r^2^ = 0.17). These results suggest that agility and COD are two different and independent abilities, both in rest and fatigue conditions (Fig 4).

This finding is also supported by the analysis between the total RA and total RCOD times, that showed a significant difference (p < 0.001; ES = 2.65 huge; r = 0.41 fair; r^2^ = 0.17).

Ratings of perceived exertions (RPE, Borg CR10) analysis reported no significant differences (p = 0.082; ES = 0.35 small) between the RA (RPE value = 7.3 ± 1.7) and RCOD (RPE value = 6.6 ± 1.9).

Finally, the Kendall rank correlation coefficient (Kendall’s tau) analysis reported a value of—0.08, showing an almost null correlation between a player’s rank order in one task and his rank in the other one.

## Discussion

To the authors’ best knowledge, this is the first study directly assessing the differences and relationships between agility and COD in fatigued conditions, and assessing how the fatigue develops in a repeated agility protocol and in a repeated COD one.

Confirming our first research question, one of the main findings is that agility and COD differ also in fatigued conditions (Fig 4), with a slight but constantly increasing effect size compared to rest conditions (ES between 1^st^ blocks = 2.02; ES between 2^nd^ blocks = 2.3; ES between 3^rd^ blocks = 2.38; ES between 4^th^ blocks = 2.7). This is a novel finding because previous studies only investigated RCOD [29–32] and RA [33, 34] separately.

Since the physical execution of the task was almost identical for the agility and COD tests (Fig 1), this increasing difference between the 2 tasks in fatigued conditions may also be explained by a possible change in decision-making performance, according to the agility definition [17]. However, it is difficult to speculate without directly measuring it, if decision-making ability improved or worsened, because the relationships between physical and cognitive efforts are extremely complex and mutual. In fact, research has shown that physical exertion caused by high-intensity physical activities may lead to a subsequent cognitive and visual-motor performance impairment [37, 38, 46], but also to an improvement, thanks to a higher hormone-related arousal and the mediating role of resource allocation [39, 47, 48]. An improvement in decision-making performance could also have counter-balanced the impairments in physical performance, meaning that impairments in agility times could have been larger. Therefore, to correctly establish if the decision-making performance and its relationship with total agility time change between rest and fatigued conditions, it is necessary to measure the decision-making times (DMTs). In rest conditions, it has been reported that the correlation between DMT and total agility time was considerable (r = 0.77), even if the DMT represented only the 3.6% of the total agility time [49]. DMTs have been measured through the video analysis of the agility test, and have been defined as the period between the stimulus appearance (light, movement of a human tester, start of a video, etc.) and the moment in which the participant plants the foot which initiates the change of direction [24, 49–51]. However, also according to one of our pilot studies, this procedure is difficult because in our design, participants made the decision and performed the COD while sprinting at high velocities, whereas in other studies they made the decision and the relative COD starting from a steady position [49, 50]. Therefore, it would be difficult to assess the DMTs precisely and reliably without considering some movement time. Finally, it must be considered that even if our task was intense, it is unlikely that just 5 minutes of exercise may have a great impact on cognitive performance over a so short period of time. This is in accordance with Chang et al. [47] and Brisswalter et al. [48], who reported that exercise must last at least 20 minutes to elicit cognitive improvements.

Concerning the development of fatigue in RCOD and RA, our results indicate that even if they are two different abilities, the trend of fatigue appearance and evolution is similar (Fig 4). The only difference between the two types of tasks is that fatigue-related impairment of performance in RCOD reached a statistical significance only at block 3, not at block 2 as in RA (always compared to the respective block 1), suggesting that fatigue may arise earlier in RA. Looking at Tab 1 and Tab 2, it is possible to see that peak decrements were 5.7% and 4% for RA and RCOD, respectively. Moreover, using the percentage decrement score (S_dec_) as proposed by Girard et al. [26], values were 7.5% for RA, and 7.3% for RCOD. As already mentioned, the advantage of combining these 2 methods to calculate fatigue is that it makes it possible to have a more accurate insight into its development, allowing to visualise its trend block by block and not only in general. RPE values also confirm that participants perceived the 2 tasks as equally fatiguing (6.6 ± 1.9 for RCOD, and 7.3 ± 1.7 for RA; ES = 0.35 small).

As already mentioned, comparing results with other studies is difficult because of the large differences between protocols (distances covered, number and angles of changes of directions, work:rest ratios, indexes of fatigue used, etc.) [26, 35]. For RA, our results are not consistent with those of Matlák et al. [33], who found no impairments during the execution of agility repetitions with a 1:3 work:rest ratio. However, the test used was different from ours, with longer distances for each repetition (~ 12.5 m vs 10 m, respectively), more changes of directions in each repetition (5 vs 1, respectively), higher number of reactions to external stimuli, different angles of each turn (0–160° vs 45°, respectively), and therefore higher number (but not intensity) of decelerations and accelerations, but less peak velocity.

Regarding RCOD, it is almost impossible to compare results, because all studies investigating RCOD used extremely different methods and protocols (distances covered, number and angles of changes of directions, work:rest ratios, indexes of fatigue used, etc.) [29–32]. However, all these cited studies [29–32] reported a decrement in RCOD performance, in line with our results.

Finally, reinforcing the idea that RCOD and RA are 2 different abilities, the almost null correlation found by the Kendall rank correlation coefficient (Kendall’s tau) means that a player may be very skilled in one of the 2 tasks, while not in the other one. This must be considered by coaches and trainers when planning a training schedule and when evaluating a player.

In conclusion, our results show that agility and COD are two different abilities also in fatigued conditions, but the development of fatigue over the execution of repeated trials follows a similar trend. Coaches and trainers may consider using a 1:5 work:rest ratio to efficiently train RA and RCOD abilities.

## Limitations

This study presents some limitations to be acknowledged. First, the Y-shaped agility test used is not the most ecological one, because it requires a simple choice (left/right) in response to a general stimulus (light). This means this kind of test is a tool with high reliability, but with low transfer to real game decision and operative abilities, which are more complex. However, we used that test because we needed a test which had to be reliable for both agility and COD with as few differences as possible between the execution of the two tasks, because this is the first study directly assessing and comparing these two abilities also in fatigued conditions (RCOD and RA). That is why we decided to use the most studied test in literature [21, 23]. Future research should now try to use more ecological tests to approach real-game situations, and that may better discriminate between high and low skilled players. A great step forward to enhance ecological validity has been made by Young and Murray [52], who developed an attacking and defending 1vs1 agility test, reporting good reliability, and demonstrating that even attacking and defending are two different abilities that require two specific tests.

## Supporting information

S1 Fig Individual RA (left) and RCOD (right) performances. The same color represents the same participant. Continuous grey line represents the mean value.
(TIF)
