# Supplementary figures and images for: Agility and change-of-direction speed are two different abilities also during the execution of repeated trials and in fatigued conditions
Source: PLoS One. 2022 Jun 10;17(6):e0269810. doi: 10.1371/journal.pone.0269810 (PMC9187116; doi:10.1371/journal.pone.0269810)

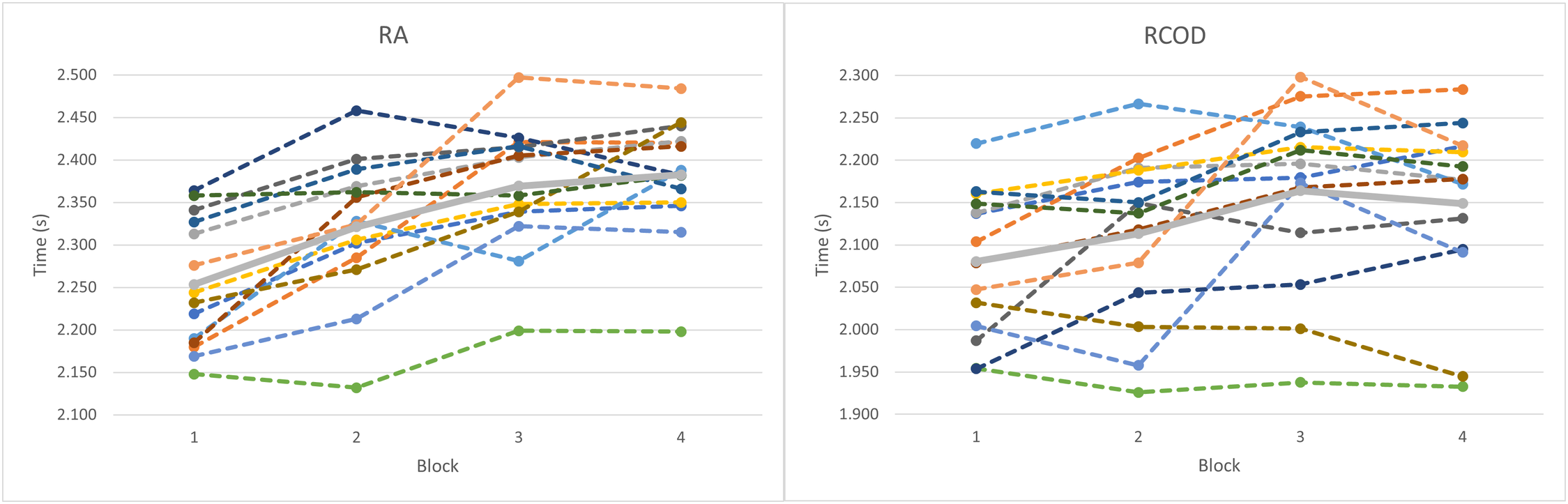

Supplement: S1 Fig — The same color represents the same participant. Continuous grey line represents the mean value. (TIF) [file pone.0269810.s001.tif]
